# Molecular pathogenic pathways in extranodal NK/T cell lymphoma

**DOI:** 10.1186/s13045-019-0716-7

**Published:** 2019-04-02

**Authors:** Sanjay de Mel, Susan Swee-Shan Hue, Anand D. Jeyasekharan, Wee-Joo Chng, Siok-Bian Ng

**Affiliations:** 1 Department of Haematology-Oncology, National University Cancer Institute of Singapore, National University Health System, 1E Kent Ridge Rd, Singapore, 119228 Singapore; 2 Department of Pathology, National University Health System, Singapore, Singapore; 3 Agency for Science Technology and Research Singapore, Institute of Molecular and Cellular Biology, Singapore, Singapore; 4 Cancer Science Institute of Singapore, National University of Singapore, Singapore, Singapore; 5 Department of Pathology, Yong Loo Lin School of Medicine, National University of Singapore, 5 Lower Kent Ridge Road, Singapore, 119074 Singapore

**Keywords:** Extranodal NK/T cell lymphoma, Molecular pathogenesis, Hallmarks of cancer

## Abstract

Extranodal NK/T cell lymphoma, nasal type (ENKTL) is an aggressive malignancy with a dismal prognosis. Although l-asparaginase-based chemotherapy has resulted in improved response rates, relapse occurs in up to 50% of patients with disseminated disease. There is hence an urgent need for effective targeted therapy, especially for patients with relapsed or refractory disease. Novel insights gleaned from high-throughput molecular and genomic profiling studies in recent years have contributed significantly to the understanding of the molecular biology of ENKTL, which exemplifies many of the hallmarks of cancer. Deregulated pro-proliferative signaling pathways, such as the Janus-associated kinase/signal transducer and activator of transcription (JAK/STAT), platelet-derived growth factor (PDGF), Aurora kinase, MYC, and NF-κB, have been identified as potential therapeutic targets. The discovery of the non-canonical function of EZH2 as a pro-proliferative transcriptional co-activator has shed further light on the pathogenesis of ENKTL. Loss of key tumor suppressor genes located on chromosome 6q21 also plays an important role. The best-studied examples include PR domain zinc finger protein 1(PRDM1), protein tyrosine phosphatase kappa (PTPRK), and FOXO3. Promoter hypermethylation has been shown to result in the downregulation of other tumor suppressor genes in ENKTL, which may be potentially targeted through hypomethylating agents. Deregulation of apoptosis through p53 mutations and upregulation of the anti-apoptotic protein, survivin, may provide a further growth advantage to this tumor. A deranged DNA damage response as a result of the aberration of ataxia telangiectasia-related (ATR) kinases can lead to significant genomic instability and may contribute to chemoresistance of ENKTL. Recently, immune evasion has emerged as a critical pathway for survival in ENKTL and may be a consequence of HLA dysregulation or STAT3-driven upregulation of programmed cell death ligand 1 (PD-L1). Immunotherapy via inhibition of programmed cell death 1 (PD-1)/PD-L1 checkpoint signaling holds great promise as a novel therapeutic option. In this review, we present an overview of the key molecular and pathogenic pathways in ENKTL, organized using the framework of the “hallmarks of cancer” as described by Hanahan and Weinberg, with a focus on those with the greatest translational potential.

## Background

Extranodal NK/T cell lymphoma, nasal type (ENKTL) is an aggressive lymphoma derived from NK cells or cytotoxic T cells. It is associated with Epstein-Barr virus (EBV) infection and is characterized by prominent necrosis, vascular damage, and cytotoxic phenotype [1]. ENKTL is prevalent in Asia, Mexico, and Central or South America and more commonly affects males than females. The EBV is present in a clonal episomal form and frequently shows a 30 bp deletion in the latent membrane protein 1 (LMP1) gene. The virus in ENKTL demonstrates a type II latency pattern characterized by the presence of EBV nuclear antigen 1 (EBNA1) and LMP1 and the absence of EBNA2 [2]. ENKTL almost always presents with extranodal disease and typically involves the upper aerodigestive tract such as the nasal cavity, nasopharynx, paranasal sinuses, and palate. Other preferential sites of involvement include the skin, soft tissue, gastrointestinal tract, and testes. Secondary lymph node involvement can occur, but the tumor rarely presents with primary nodal disease, in contrast to EBV-positive nodal T cell or NK cell lymphomas [3]. Morphologically, ENKTL demonstrates diffuse and permeative lymphoid infiltrate which is often associated with ulceration, necrosis, and angiodestructive growth pattern. The tumor cells display a broad spectrum of cytology ranging from small to large and anaplastic cells. Immunophenotypically, the malignant cells are positive for cCD3 (or CD2), CD56, cytotoxic markers (such as TIA1, granzyme B, and perforin), and EBV [1, 2].

The genomics of T and NK cell lymphomas have been an active field of research in recent years. Novel insights gleaned from high-throughput molecular and genomic profiling studies have contributed significantly to the understanding of peripheral T and NK cell lymphomas. The identification of a multistep oncogenic pathway which involves epigenetic deregulation related to TET2, DNMT3, or IDH2 mutations and mutations affecting genes related to TCR signaling pathway in nodal lymphomas of follicular helper T cell origin has been a major advancement [4]. Similarly, the uncovering of unique gene expression profiles with the identification of dysregulated signaling pathways [5–7], as well as the characterization of the mutational landscape via mutational profiling techniques in ENKTL, is providing new insights into the pathogenesis of this disease [8].

There is evidence that the functional deregulations resulting from the genetic alterations may contribute to the oncogenesis or maintenance of some characteristics of the malignant phenotype. As normal cells evolve progressively to a neoplastic state, they acquire hallmark traits which enable them to become tumorigenic and eventually undergo malignant transformation [9]. Immune evasion is another hallmark of cancer, which allows malignant cells to avoid detection by the host immune system, thereby evading eradication. The ability of tumor cells to escape immune surveillance is based on cellular and molecular characteristics of the tumor microenvironment [10]. Pembrolizumab, an anti-PD-1 antibody, has recently been demonstrated to be effective in a series of patients with advanced ENKTL, supporting the role of immune checkpoint molecules and immune evasion in the development of this aggressive tumor [11].

**Fig. 1:**
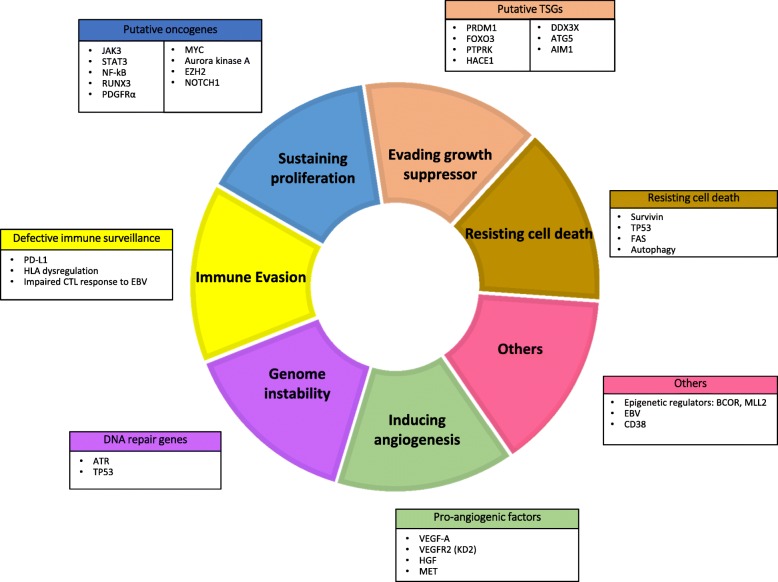
Schematic overview of deregulated genes in extranodal NK/T cell lymphoma, nasal type grouped by known mechanisms or functions (inspired by the hallmarks of cancer by Hanahan and Weinberg)

**Fig. 2:**
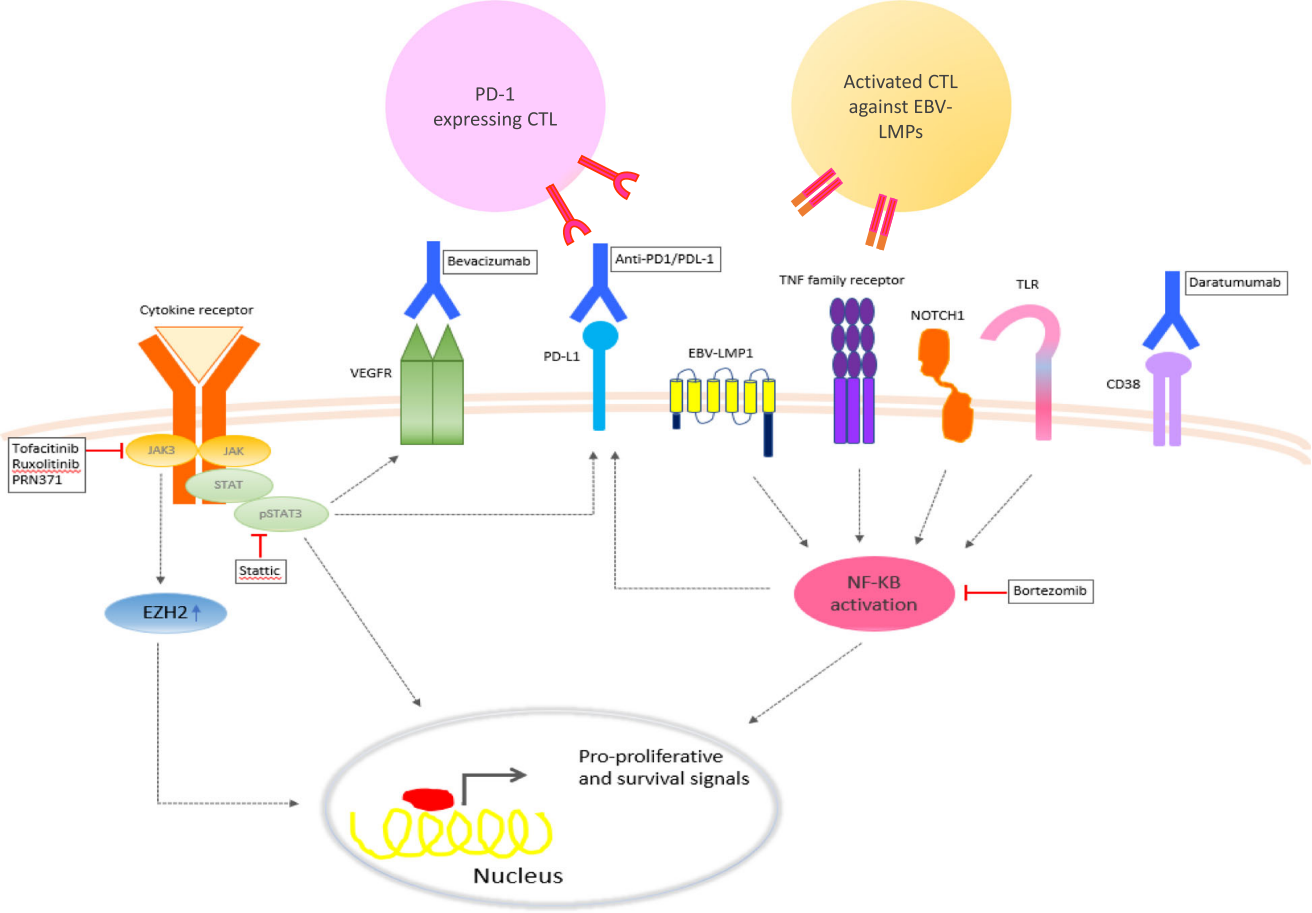
Targeted therapies with greatest clinical significance based on biological knowledge in ENKTL. Extranodal NK/T cell lymphoma, nasal type (ENKTL) frequently show oncogenic activation of JAK/STAT and NF-κB pathways that promote proliferation and survival of the lymphoma cells. These signaling pathways can be targeted by a variety of small molecule inhibitors. Antibody drugs targeting a number of overexpressed membrane proteins, such as PD-L1, CD38, and vascular endothelial growth factor receptor (VEGFR), are being evaluated in clinical trials

In this review, we aim to summarize key molecular alterations in ENKTL, organized using the framework of the “hallmarks of cancer” as described by Hanahan and Weinberg [9] (Fig. 1), with an emphasis on those with potential translational impact (Fig. 2). The biological and clinical significance of these pathways is summarized in Table 1. The most promising novel therapeutic options targeting these pathways are summarized in Table 2.

**Table 1:** The key pathogenic pathways in ENKTL are described with a summary of the specific molecular/genetic abnormalities involved in each pathway. Evidence for each pathway as a therapeutic target is indicated where applicable

Mechanism of Lymphomagenesis (Hallmarks of cancer)	Specific pathway / target	Role in Lymphoma Biology	References	Therapeutic Significance	References
Sustaining proliferative signaling	JAK/STAT	Upregulated via mutation or phosphorylation	Huang et al. [5, 80]	Anti-tumor activity of JAK-3 and STAT-3 inhibition in pre-clinical / in vitro models. Clinical trials evaluating JAK inhibitors in ENKTL ongoing.	Nairismagi et al. [24]
					Sim et al. [19]
	RUNX3	Upregulated and has oncogenic role promoting proliferation and survival in ENTKL.	Selvarajan et al. [44]	MYC inhibition in vitro leads to down-regulation of RUNX3 and apoptosis, suggesting MYC as potential therapeutic target.	Selvarajan et al. [44]
	EZH2	Upregulated and functions as a transcriptional co-activator via a non-canonical pathway.	Yan et al. [27]	Targeting EZH2 using a PCR2 inhibitor induces apoptosis in ENKTL.	Yan et al. [28]
	NF-kB	Upregulated and promotes survival and proliferation.	Huang et al. [5, 80]	Bortezomib in ongoing early phase clinical trials for ENKTL.	Liu et al. [32]
			Ng et al. [7, 41]		Tang et al [36]
					Chen et al. [35]
	AURKA	Upregulated, promotes cell proliferation.	Iqbal et al. [6]	In vitro inhibition of AURKA induced apoptosis	Iqbal et al. [6]
			Ng et al. [7, 41]		
	PDGFRα	Upregulated. Mediates migration, proliferation and cell survival.	de Mel et al. [162]	Potential therapeutic target for tyrosine kinase inhibitors.	Huang et al. [5, 80]
	NOTCH	Upregulated in ENKTL, involved in developmental processes and cancer.	Huang et al. [5, 80]	Potential therapeutic target for NOTCH inhibitors.	Aster et al. [163]
	CDK2, HSPCA	Upregulated. Promotes proliferation and survival of cancer cells.	Zhang et al. [164]	N/A	N/A
	DDX3X	RNA helicase, loss of function mutations lead to cell cycle progression and activation of other pro-proliferative pathways	Jiang et al [18]	N/A	N/A
Evading Growth Supressors /Resisting Cell Death	Survivin	Upregulated in the majority of ENKTL. Inhibits apoptosis.	Ng et al. [7, 41]	Survivin inhibition in vitro induced apoptosis, suggesting potential therapeutic role.	Ng et al. [7, 41],
			Ng et al. [165]		de Mel et al. [162]
	P53	Upregulated (e.g. by mutation). Inhibits apoptosis.	Ng et al. [7, 41]	N/A	N/A
			Quintanilla Martinez et al. [66]		
	BIRC1, IL-1A, TNFRS10D	Upregulated, inhibits apoptosis.	Zhang et al. [164]	N/A	N/A
	PTPRK	Frequently deleted and hypermethylated. Re expression suppressed proliferation and induced apoptosis. Precise function under evaluation		N/A	N/A
	PRDM1	Frequently deleted in and re expression leads to cell growth. Functional role under investigation.		N/A	N/A
	FOXO3	Frequently deleted in apoptosis induced by re-expression. Function under investigation		N/A	N/A
	HACE1	Encodes E3 ubiquitin ligase, frequently deleted and hypermethylated. Function Under investigation		N/A	N/A
	ATG5	Candidate tumour suppressor gene awaiting evaluation of function.		N/A	N/A
	AIM1	Candidate tumour suppressor gene awaiting evaluation of function.		N/A	N/A
	Autophagy pathway	Beclin 1 under-expression is associated with a worse prognosis	Huang et al. [5, 80]	Response to HDAC inhibition in combination with bortezomib in two patients with RR ENKTL.	Tan et al. [81]
Immune Evasion	PD-L1	Upregulated. Involved in immune evasion.	Ng et al. [3, 116]	Patients with relapsed ENKTL showed response to pembrolizumab, an antibody against PD1.	Kwong et al. [11]
			de Mel et al. [162]		
Genomic Instability/Deregulated DDR	ATR	Deregulation (e.g. deletion) resulting in abnormal DNA damage response.	Liu et al. [123]	N/A	N/A
Angiogenesis	VEGF	Upregulated. Promotes tumour vascularization and growth.	Jørgensen et al. [127], de Mel et al. [162]	Potential therapeutic target.	Jørgensen et al. [127]
Other Mechanisms and Targets					
Epigenetic Deregulation	Promoter Hypermethylation	Widespread promoter hypermethylation leading to down regulation of tumor suppressor genes.	Kucuk et al. [20]	N/A	N/A
	BCOR	Interacts with HDAC family. Role in ENKTL under evaluation	Huynh et al. [157]	N/A	N/A
	MLL2	Histone methyltransferase. Role in ENKTL under evaluation	Milne et al. [158]	N/A	N/A
	miR-150	Downregulated of miRNAs in ENKTL. Targets of these miRNAs include genes in critical pathways such as p53, MAPK and EZH2	Ng et al. [7, 41]	N/A	N/A
	miR-101				
	miR-26a				
	miR-26b				
	miR-28-5				
	miR-363				
	miR-146				
	miR-21	Upregulated and have a pro-oncogenic function	Yamanaka et al. [59]	N/A	N/A
	miR-155				
	miR-146a	Downregulated, associated with poor prognosis	Paik et al. [104]	N/A	N/A
	miR-221	Upregulated, associated with poor prognosis.	Guo et al. [166]	N/A	N/A
EBV Mediated Mechanisms	EBV lytic genes (BHRF1, BKRF3, BZLF1)	Upregulated. Potential pathogenic role in ENKTL . BHRF1 may have anti-apoptotic role due to sequence homolog to human BCL-2.	Zhang et al. [164]	N/A	N/A
CD38	CD38	Upregulated. Exact role unknown but associated with poorer prognosis.	Hu et al. [167]	Good in-vitro efficacy of daratumumab and one case report documenting complete response.	Mustafa et al. [153]
					Hari et al. [154]

Abbreviations: *ENKTL* Extranodal NK Tcell lymphoma, *EBV* Epstein Barr Virus, *HDAC* Histone deacetylase, *RR* Relapsed Refractory, *N/A* No available data to support a therapeutic role at present

**Table 2:** The most promising novel therapeutic options in ENKTL are summarized. The biological basis for targeting these pathways along with available clinical data are shown

Signaling pathway or therapeutic target	Biological basis for selection as a therapeutic target	Clinical data	References
JAK3	JAK3 mutations are frequent in ENKTL. JAK3 inhibition is shown to have potent anti-tumor activity in pre-clinical models	Clinical trials evaluating JAK inhibitors in ENKTL are in progress. (NCT02974647)	Sim et al. [19] Narisimagi et al. [24]
STAT-3	STAT3-mutant ENKTLs are sensitive to STAT3 inhibition in vitro.	Not available.	Sim et al. [19]
NF-κB	NF-κB upregulation is an important event in ENKTL pathogenesis.	Bortezomib is being evaluated in early phase clinical trials for ENKTL	Chen et al. [35] Tang et al. [36]
CD38	CD38 is upregulated in ENKTL. Daratumumab has good in vitro efficacy.	One case report documenting complete response in a relapsed refractory patient.	Mustafa et al. [153] Hari et al. [154]
PD-1	PD-L1 is upregulated in ENKTL.	Early clinical trials show potent single-agent activity of anti-PD-1 therapy in relapsed, refractory ENKTL.	Kwong et al. [11]

## Pro-proliferative signaling pathways

Sustained proliferative signaling is one of the fundamental characteristics of cancer [9]. This is achieved through numerous mechanisms including but not limited to mutations leading to aberrant pro-proliferative signaling, escape from negative feedback mechanisms that attenuate proliferation, and evading growth suppressors [9]. The last decade has seen a marked improvement in our understanding of pro-proliferative mechanisms underlying ENKTL which will be elaborated on in this section. It is important to appreciate that the pathways that lead to the proliferation of cells of immune origin are distinct from those driving epithelial cancers.

### JAK/STAT and related pathways

The Janus kinase/signal transducer and activator of transcription (JAK/STAT) pathway plays a crucial role in the pathogenesis of ENKTL through its pro-proliferative function [12, 13]. Gene expression profiling (GEP) data has revealed the upregulation of JAK/STAT pathway genes in ENKTL compared to normal NK cells [5, 14]. JAK3, STAT3, and STAT5B mutations leading to the constitutive activation of the JAK/STAT pathway have been reported in ENKTL. However, their mutational frequency varies between studies and ranges from 0 to 35% [12, 14–17]. Other studies have reported a lower frequency of JAK3 mutations and more frequent JAK3 phosphorylation in ENKTL instead [15, 17–19]. Bouchekioua et al. reported JAK3 phosphorylation in 87% of cases while only 20% of these were a result of activating mutations in the pseudokinase domain [12]. Similarly, STAT3 and STAT5B mutations are not common in ENKTL, while STAT3 phosphorylation was found in the majority of cases [5, 13, 20].

The PTPRK gene encoding the receptor-type tyrosine-protein phosphatase κ is located on chromosome 6q, a region commonly deleted in ENKTL [21]. PTPRK is known to dephosphorylate phospho-STAT3, leading to its inactivation [21]. Hence, underexpression of PTPRK due to deletions and aberrant promoter hypermethylation of PTPRK has been proposed to cause constitutive STAT3 activation resulting in proliferation and ENKTL lymphomagenesis [21]. PTPRK downregulation was shown to be associated with advanced stage disease and an inferior outcome in patients treated with the steroid, methotrexate, ifosphamide, l-asparaginase, and etoposide (SMILE) protocol [21]. STAT3 activation may be indirectly targeted by increasing the expression of PTPRK. As hypermethylation of the PTPRK promoter leads to its underexpression, demethylating agents, such as 5-azacytidine, may be able to restore PTPRK expression resulting in the downregulation of STAT3 activity [21].

GEP studies have demonstrated the upregulation of interleukin-2 (IL-2), IL-10, interferon gamma receptor 1 (IFNGR1), and inhibin beta A (INHBA) in ENKTL [22]. The binding of IFN-γ to IFNGR1 was shown to result in the activation of the JAK/STAT pathway adding to the multiple modalities of JAK/STAT dysregulation in ENKTL [23].

These data suggest that constitutive activation of the JAK/STAT pathway in ENKTL arises through diverse mechanisms including mutations and aberrant phosphorylation. Importantly, the JAK/STAT pathway is a potential therapeutic target in ENKTL. Sim et al. reported JAK3H583Y and JAK3G589D, two novel JAK3 mutations, which had more lymphomagenic potential and were also more sensitive to the JAK inhibitor tofacitinib [19]. A selective inhibitor of JAK3 (PRN371) has recently been demonstrated to have a more potent anti-tumor activity than tofacitinib in a xenograft model of ENKTL with a JAK3 mutation [24]. It is noteworthy that STAT3-mutant ENKTL cells were sensitive to the STAT3 inhibitor, Stattic, but not to tofacitinib [19]. Based on these findings, future clinical trials would benefit from the mutational profiling data of the tumor to guide therapeutic interventions. Ruxolitinib, a JAK1/2 inhibitor approved for myelofibrosis, is now being investigated in phase II clinical trials for patients with relapsed ENKTL (NCT02974647) [25].

The histone methyltransferase EZH2 is a component of the polycomb repressive complex 2 (PRC2), which is involved in epigenetic regulation [26]. EZH2 has recently been shown to function as a transcriptional co-activator via a non-canonical pathway that is independent of its enzymatic activity [27]. JAK3-mediated phosphorylation of EZH2 promotes this switch from histone methyltransferase to transcriptional co-activator, resulting in the upregulation of genes involved in cell cycle regulation, DNA replication, invasiveness, and stemness [27, 28]. This non-canonical function is the mechanism by which EZH2 mediates the proliferation and survival in ENKTL [27]. Inhibition of EZH2 enzymatic activity is currently actively evaluated in clinical trials in diffuse large B cell lymphoma and follicular lymphoma, where mutations in EZH2 affect its enzymatic and canonical function [29]. In fact, these inhibitors do not work in ENKTL cell lines as the enzymatic function is not required for EZH2 oncogenic functions. Strategies to downregulate EZH2 protein are needed for the therapeutic effect in ENKTL. Indeed, much of the oncogenic activity downstream of JAK/STAT in ENKTL is mediated by non-canonical EZH2 as much of the effect of JAK inhibition can be rescued by an EZH2-phosphomimetic mutant. One of the important proliferative genes activated by EZH2 is cyclin D1. Indeed, the combined inhibition of JAK2 and CDK4/6 with ruxolitinib and LEE001, respectively, produces synergistic inhibition of ENKTL growth and survival [30].

A better understanding of the mechanisms behind these aberrations is critical for the development of an effective targeted therapy.

### Nuclear factor kB pathway

Nuclear factor κB (NF-κB) is a transcription factor involved in pro-proliferative signaling in a variety of lymphoid malignancies [31]. GEP studies have suggested an increased expression of NF-κB-related genes in ENKTL, although this finding was not consistent across studies [5–7]. NF-κB activation in ENKTL was proposed to occur via the non-canonical pathway in ENKTL by Liu and colleagues although Ng et al. showed more prominent utilization of the canonical pathway [7, 32]. Further studies are required to elucidate the mechanisms of NF-κB activation in ENKTL. Treatment of ENKTL cell lines with NF-κB inhibitors resulted in the induction of apoptosis, supporting the hypothesis that this pathway plays an important role in ENKTL [32].

NF-κB has recently been implicated in the pathogenesis of hemophagocytosis, which is a leading cause of death in ENKTL patients [33]. Evolutionarily conserved signaling intermediate in Toll pathway (ECSIT) is a cytoplasmic protein which plays an important role in Toll-like receptor 4 (TLR4)-mediated signaling to activate NF-κB [34]. Wen and colleagues reported a hotspot mutation (ECSIT-V140A) in 19% of ENTKL cases. These patients had a higher incidence of hemophagocytic syndrome (HPS), a more aggressive disease, and a worse prognosis [33]. The study revealed that ENKTL patients frequently harbor the ECSIT-T419C somatic mutation which activates the NF-κB pathway, thereby inducing the release of pro-inflammatory cytokines (including TNF-α and IFN-γ) and promoting macrophage activation and hemophagocytosis. Two ENKTL patients with HPS who had the ECSIT-V140A mutation showed a resolution of HPS on treatment with thalidomide and dexamethasone. The proposed mechanism of action of thalidomide in this setting was by preventing NF-κB from binding to the promoters of its target genes, IFN-γ and TNF [33]. Further studies are necessary to determine the frequency and role of ECSIT-V140A in ENKTL as well as how this may be adopted as a therapeutic target.

DDX3X is an RNA helicase gene which is mutated in 50% of ENKTL [18]. Inactivating DDX3X mutations was shown to result in cell cycle progression and transcriptional activation of the NF-κB pathway [18]. DDX3X was proposed to interact with both pro-proliferative and anti-apoptotic pathways in ENKTL and will be discussed further in the “p53” section [18].

Although targeting NF-κB through bortezomib-based regimens has been attempted in ENKTL, these studies were based on small patient numbers and larger clinical trials are required for a more thorough assessment of efficacy and safety [35, 36].

### C-MYC

Deregulation of the oncogene C-MYC is known to play a pivotal role in the pathogenesis of several cancers including lymphoma [37–39]. GEP studies have shown that C-MYC is overexpressed in ENKTL [7]. Although translocations involving the MYC gene have not been demonstrated in ENKTL, the overexpression of MYC together with the anti-apoptotic protein BCL2 by immunohistochemistry has been proposed as an adverse prognostic factor [40]. As C-MYC is a transcriptional target of EBNA2 and LMP1, it has been hypothesized that C-MYC upregulation in ENKTL is mediated by EBV [7]. C-MYC activation results in significant transcriptional deregulation, leading to the upregulation of MYC transcriptional targets, such as EZH2 and RUNX3 [41].

The overexpression of MYC is proposed as one of the mechanisms behind miRNA downregulation in ENKTL [41]. MYC-induced downregulation of microRNAs miR-26a and miR-101 leads to the upregulation of their target EZH2 in ENKTL which has pro-proliferative functions as discussed in the “Pro-proliferative signaling pathways” section [41, 42]. Although MYC has been evaluated as a therapeutic target in other lymphomas, this strategy is yet to be explored in ENKTL [43].

### RUNX3

Runt-domain transcription factor 3 (RUNX3), a master transcriptional regulator in major developmental pathways, has been shown to be transcriptionally regulated by C-MYC [44]. RUNX3 has not traditionally been recognized as a pro-proliferative oncogene in lymphoma. In cytotoxic T and NK cells, RUNX3 mediates the transcriptional activation of genes involved in proliferation and effector function, including interferon gamma, perforin, and granzyme B [45]. RUNX3 expression is upregulated in ENKTL, as well as in aggressive B and T cell lymphomas [44]. SiRNA-induced silencing of RUNX3 resulted in increased apoptosis and reduced cell proliferation in ENKTL cell lines, suggesting an oncogenic role for RUNX3 in ENKTL [44]. MYC inhibition using a novel small molecule, JQ1, resulted in the downregulation of RUNX3 transcripts, supporting the hypothesis that RUNX3 is a transcriptional target of MYC [44].

### PDGF pathway

Platelet-derived growth factor receptor alpha (PDGFRα) is a receptor tyrosine kinase known to mediate the proliferation and survival in hematopoietic malignancies [46]. PDGFRα, as well as its phosphorylated form, is overexpressed in ENKTL, indicating activation of this pathway [5]. The cause of PDGFRA upregulation in ENKTL requires further study as mutations in this gene have not been reported in ENKTL [5]. Imatinib, a tyrosine kinase inhibitor approved for chronic myeloid leukemia, induced growth inhibition in a PDGFRα-expressing ENKTL cell line, suggesting potential as a therapeutic target [5].

### NOTCH1 pathway

The NOTCH signaling pathway has been reported to mediate pro-proliferative signaling in lymphoid malignancies [47]. GEP studies have shown an upregulation of NOTCH pathway genes in ENKTL [5, 6]. The role of NOTCH signaling in the survival of ENTKL was supported by NOTCH inhibitors inducing significant growth inhibition in two NK cell lines [6]. Further studies are required to delineate the basis of NOTCH upregulation in ENKTL and to better evaluate the role of NOTCH inhibition as a therapeutic strategy.

### Aurora kinase pathway

Aurora kinase A (AURKA) is a serine/threonine kinase that contributes to cell cycle regulation [48]. Although the main role of AURKA is in promoting mitosis, it has recently been shown to participate in cell proliferation through its interaction with *MYC* and *WNT* signaling, while inhibiting *TP53* [49–52]. AURKA mRNA and protein have been shown to be upregulated in ENKTL patient samples and cell lines [6, 7]. The location of the gene encoding AURKA at a frequently (> 50%) amplified locus in ENKTL may explain these findings [6, 53]. Treatment of ENTKL cell lines with a small molecule AURKA inhibitor (MK-8745) resulted in a significant increase in apoptosis and cell cycle arrest, suggesting potential as a therapeutic target [6]. Furthermore, AURKA overexpression is associated with resistance to taxane chemotherapy and AURKA inhibition sensitizes tumors to paclitaxel [49].

### Others pro-proliferative pathways

Copy number analysis of ENKTL has revealed chromosomal gains involving the following regions which result in the overexpression of genes promoting cell cycle proliferation:1q (*CDCA1*, *NEK2*), 2q (*E2F6*), 7q (*RHEB*), 17q (*CDC27*), and 20p (*DSTN*) [53]. The importance of each of these chromosomal gains on ENKTL pathogenesis requires further evaluation as does their interaction with the other pro-proliferative pathways above. Other genes which may drive proliferation and are upregulated in ENKTL cell lines include cyclin-dependent kinase 2 (CDK2), a regulator of cell cycle progression, and heat shock 90 kDa protein 1-alpha (HSPCA), which plays a role in protein folding and cell survival [54]. Data on the precise role and importance of these genes in the pathogenesis of ENKTL is limited.

MicroRNAs (miRNA) are a class of short, non-coding RNA which plays an important role in the regulation of gene expression [55]. On the whole, microRNAs negatively regulate gene expression and their role in ENKTL has been recently reviewed [56–58].miRNA deregulation has been reported to affect pro-proliferative pathways such as AKT and MAPK. Specifically, miR-21 and miR-155 are overexpressed in ENKTL, resulting in the deregulation of the AKT pathway [41, 59]. Chang et al. further demonstrated that miR-155 regulates lymphangiogenesis in ENKTL by targeting brahma-related gene 1 (BRG1). In addition, targeting miR-155 inhibited the growth of ENKTL xenografts as well as tumor-associated lymphangiogenesis in vivo [60].

Among the pro-proliferative pathways discussed, JAK/STAT and NF-κB are the best studied and likely to have the most biological relevance in ENKTL. Drugs targeting these pathways have made significant progress in terms of clinical development and have the greatest translational potential.

## Resisting cell death

Resisting cell death is one of the hallmarks of cancer, which is established as a mechanism of cell survival [9, 61]. Evasion of apoptosis is also emerging as a key player in the pathogenesis of ENKTL. Apoptotic deregulation in ENKTL occurs via multiple mechanisms which will be discussed in this section.

### Survivin

Survivin is an anti-apoptotic protein which inhibits caspase activation and was shown to be overexpressed by GEP in 97% of ENKTL [7, 62]. The overexpression of survivin is associated with aggressive behavior in other malignancies and may contribute to the relative resistance of ENKTL to cytotoxic chemotherapy [7, 63]. In vitro studies using a survivin inhibitor, terameprocol, led to a significant increase in apoptosis and decreased viability of tumor cells, thus presenting a potential therapeutic opportunity [7]. Although there is a promising in vitro data, survivin inhibition is yet to be explored in clinical trials. It is noteworthy that survivin is one of the target genes of NF-κB and its upregulation may be related to the activation of NF-κB in ENKTL [7]. Upregulation of survivin has also been linked to TP53 loss in other malignancies; this may also be the case in ENKTL based on TP53 deregulation in ENKTL discussed below [7, 64].

### p53

Mutations in the key tumor suppressor p53 are common in cancer, [65] often leading to an anti-apoptotic phenotype which promotes cell survival. Deregulation of p53 in ENKTL may be attributed to mutations, EBNA1-induced p53 degradation and/or mutations in apoptotic proteins downstream of p53, such as FAS [66–68]. p53 mutations are present in up to 63% of cases of ENKTL by Sanger sequencing although they were detected at a lower frequency when assessed by next-generation sequencing [69]. The majority of mutations occur in the functional domain and were found to have a dominant negative function [69]. p53 mutations were also associated with advanced stage disease, suggesting this represents a secondary rather than an initiating oncogenic event in ENKTL [66, 70–72]. p53 mutations were shown to have a more adverse impact when they occurred concurrently with DDX3X mutations in ENKTL [18]. Furthermore, the occurrence of p53 and DDX3X mutations together was rare, suggesting some functional overlap [18]. Further studies are required to clarify the precise function of DDX3X and its relationship with p53 in ENKTL. Beyond mutations, GEP studies demonstrated that the upregulation of p53 in ENKTL was associated with the deregulation of genes which are normally controlled by p53, indicating a functional defect of p53. miRNA deregulation has also been reported to play a role in the deregulation of p53 along with other cell cycle regulatory genes [41]. The miRNAs responsible include miR-150, miR-101, miR-26a, miR-26b, miR-28-5, miR-363, and miR-146, which are generally downregulated in ENKTL compared to normal NK cells [41, 73]. Restoration of p53 function is an attractive therapeutic target which is being explored actively in other cancers; however, there is currently no data to support this in ENKTL [74].

### Autophagy and metabolic reprogramming

Alterations of cellular metabolism and homeostasis are a key feature of malignancy [9, 75]. Autophagy is a physiologic mechanism used by cells to breakdown and recycle cytoplasmic proteins and dysregulated organelles [76]. The role of autophagy in cancer is complex. Tumor cells can use autophagy to optimize their metabolism and survive in a stressed, nutrient-poor environment [77]. Alternatively, autophagy can result in cancer cell death through cross talk with the apoptotic pathway [78]. *ATG5* is a gene involved in autophagy pathway that, as mentioned in the “Other deleted genes within chromosome 6q21” section, was identified as a candidate TSG in ENKTL not only because it was located within the frequently deleted region of chromosome 6p21, but also because its expression was downregulated in tumor samples. However, no tumor suppressive effect was observed in in vitro functional analysis involving forced re-expression of the gene in 6q-deleted NK cell lines [79], raising controversy on its importance in the pathogenesis of NK cell neoplasms. Beclin 1, another protein known to be important for effective autophagy, is expressed in ENKTL [78, 80]. ENKTL patients with lower Beclin 1 expression were shown to have more aggressive disease and a worse outcome [80].

Histone deacetylase (HDAC) inhibitors have been shown to induce autophagy via suppression of mammalian target of rapamycin (M-TOR) signaling [76]. The combination of bortezomib and panabinostat was safe and effective in two ENKTL patients enrolled in a phase II trial for relapsed PTCL [81]. Another pilot study of a single-agent romidepsin in relapsed ENKTL was however closed early due to EBV reactivation in three patients [82]. The safety and efficacy of HDAC inhibition and the precise role of autophagy in ENKTL therefore require further evaluation.

## Evading growth suppressors

Products of tumor suppressor genes (TSGs) are known to play a critical role in counteracting growth-promoting signals induced by the activation of oncogenes. In ENKTL, as in many cancer types, inactivation of TSG function appears to contribute to the oncogenic mechanism by removing the negative regulatory effects on cell growth and proliferation. This evasion of growth suppression may occur through a variety of genetic and epigenetic mechanisms, such as loss-of-function mutations (i.e., deletion, truncating, or missense mutations) and promoter hypermethylation of TSGs. Earlier genomic studies using comparative genomic hybridization and GEP in ENKTCL primary tumors and cell lines have identified several putative TSGs with potential contribution to the pathogenesis of ENKTL [5, 7, 21, 53, 79, 83–86]. However, very few studies have actually demonstrated the tumor suppressive effects of these genes with functional analyses. Of all the proposed candidate TSGs, PRDM1, FOXO3, and PTPRK appear to be the most studied putative TSGs implicated in NK cell neoplasms that are supported by in vitro functional work [21, 79, 86].

### PRDM1

*PRDM1* encodes a transcription repressor Blimp1, which has been firmly established in earlier studies to be essential for the terminal differentiation of B cells into antibody-secreting plasma cells. [87–89] *PRDM1*/Blimp1 expression has also been found in T [90, 91] and NKcells [5, 41, 53] and is believed to have a role in the regulation of T and NK cell homeostasis [86, 90, 91].

The role of *PRDM1* as a TSG in lymphoma has been examined in several studies. Using in vivo mouse models, *PRDM1*/Blimp1 has been demonstrated to act as a tumor suppressor in activated B cell-like diffuse large B cell lymphoma (ABC-DLBCL) [92, 93]. As *PRDM1* is one of the candidate genes found within the frequently deleted chromosomal region 6q21-6q25 in ENKTL [53, 86], this raises a reasonable consideration of its potential tumor suppressor role in NK neoplasms as well. Indeed, using a combination of genomic and functional analyses, several independent groups have produced converging evidence supporting a tumor suppressor function of *PRDM1*/Blimp1 in ENKTL [53, 79, 86]. Using in vitro functional assays, these investigators found that re-expression of *PRDM1* in *PRDM1*-null NK cell lines led to the suppression of cell growth through cell cycle arrest and/or apoptosis [79, 86]. In addition to monoallelic deletions, truncating mutations and promoter-associated CpG island hypermethylation have also been observed in NKcell lines and primary tumors [53, 86, 94], supporting the notion that *PRDM1* is a frequently inactivated TSG in ENKTL. While the transcriptional targets of *PRDM1* in ENKTL are still under investigation, one study has identified several cancer-associated long non-coding RNAs, such as MIR155HG and TERC, as potential targets of *PRDM1* [95]*.*

### FOXO3

Another candidate TSG within the frequently deleted region of 6q21 is *FOXO3*. *FOXO3* is a member of the forkhead family of proteins that possess the forkhead box domain, and its role as a TSG has been recognized through functional studies on other tumors such as breast [96] and pituitary [97] cancers. In keeping with its frequently deleted status, downregulation of *FOXO3* expression has been shown in NK cell primary tumors as well as neoplastic cell lines [53, 79]. The tumor suppressive function of *FOXO3* was demonstrated in in vitro functional study in NK cell lines where forced expression of *FOXO3* led to the suppression of cell line proliferation through the induction of apoptosis and cell cycle arrest [79] implicating its role in the pathogenesis of NK cell neoplasms. In fact, Karube et al. went on further to show NK cell lineage-specific growth inhibition of *FOXO3* expression in association with low AKT activity, suggesting the possibility of using *FOXO3* gene therapy to achieve minimal side effects in the treatment of NK cell neoplasms [98].

### Other deleted genes within chromosome 6q21

*HACE1*, which encodes for an E3 ubiquitin ligase, is a well-known TSG in many other tumors [99]. Its potential tumor suppressive role was being investigated in ENKTL because this gene also appears to sustain frequent genomic deletions as well as promoter hypermethylation leading to its downregulated expression in ENKTL [5, 100]. However, in vitro functional work revealed no obvious tumor-suppressing effect of *HACE1* on NK cell proliferation [79].

*PTPRK* encodes for receptor-type tyrosine-protein phosphatase κ and is believed to mediate tumor suppressive function by interacting with and inactivating STAT3 [21], as mentioned in the “Pro-proliferative signaling pathways” section. Indeed, consistent with the proposed tumor suppressive properties of *PTPRK*, in vitro functional assays on cell lines showed that *PTPRK* re-expression suppressed cellular proliferation and significantly increased apoptotic rate, whereas the knockdown of *PTPRK* led to the abolishment of the oncosuppressive effects by restoring the cellular proliferation rate [21].

*ATG5* and *AIM1* were identified as candidate TSGs in ENKTL through array CGH analysis and GEP [5, 53]. However, the proposed TSG function of these genes in NK cell neoplasms has not been further supported by functional analyses.

### Other candidate TSGs discovered through mutational profiling

As mentioned in the “p53” section, p53 is an essential tumor suppressor implicated in a variety of malignancies. Early studies reported *TP53* mutations in ENKTL cases, which were observed in more than half of all cases [14, 18, 70–72, 101], and have been associated with worse prognosis and higher disease stage [18, 66], implicating the involvement of this tumor suppressor (albeit probably at a later stage) in ENKTL as well, among many other malignancies.

*DDX3X* is an RNA helicase gene which is mutated in 50% of ENKTL, implicating its involvement in the molecular pathogenesis of this aggressive neoplasm. Mutations in *DDX3X* are believed to decrease its RNA-unwinding activity, leading to the loss of suppressive function on cell cycle progression in NK cells and transcriptional activation of NF-κB and MAPK pathways [18].

### Tumor suppressor genes downregulated through promoter hypermethylation

Among TSGs downregulated due to promoter hypermethylation are *BCL2L11* (*BIM*), *DAPK1*, *PTPN6* (*SHP1*), *TET2*, *SOCS6*, and *ASNS* [102]. Restoration of BIM (a pro-apoptotic protein) and SOCS6 expression resulted in the chemosensitization and growth inhibition of ENKTL cell lines [102]. However, downregulation of ASNS (asparagine synthetase) expression was associated with increased sensitivity to l-asparaginase treatment. These data suggest that promoter hypermethylation leads to critical changes in the biology of malignant NK cells which can be potentially targeted through hypomethylating agents and asparaginase [102]. Using a methylation-specific polymerase chain reaction (MSP), Siu et al. demonstrated promoter methylation of another group of tumor suppressor genes (*p15*, *p16*, *p73*, *hMLH1*, and *RARβ)* in ENKTL [103]. The majority of cases (94%) showed methylation and downregulation of the *p73* gene. Treatment with the hypomethylating agent 5-azacytidine was able to restore p73 gene expression [103]. Intriguingly, MSP performed on histologically negative bone marrow and oropharyngeal biopsies of a patient with ENKTL yielded positive results [103]. Furthermore, the primary tumor and the bone marrow biopsy showed different methylation patterns, indicating clonal evolution. These data suggest that promoter methylation may be present before histologically and clinically overt lymphoma manifests. The possibility of promoter methylation being used for diagnostic purposes is a worthy consideration although specificity and sensitivity of the assay will need to be assessed. Although hypomethylating agents maybe an attractive therapeutic option for ENKTL, a full understanding of the epigenomic landscape of ENKTL is required prior to clinical translation. A potential concern is that hypomethylating agents may desensitize ENKTL cells to asparaginase by upregulating ASN [102].

### Other targets with tumor suppressor functions

miRNA-146a has been showed to downregulate NF-κB activity via targeting TRAF6 and functions as a tumor suppressor in ENKTL. In addition, low miR-146a expression is associated with chemoresistance in ENKTL patients [104]. Further studies are necessary to evaluate the clinical utility of miRNA expression as prognostic and/or predictive markers.

In summary, most candidates of TSGs that have been identified can be inactivated by deletion and by promoter hypermethylation. Among these, perhaps PRDM1, FOXO3, and PTPRK are the most comprehensively evaluated thus far with supporting functional analyses performed specifically on NK neoplastic cell lines. To restore the function of inactivated TSGs as a therapeutic strategy, however, remains a challenging task. In this respect, the most feasible therapeutic option probably relies on reverting the methylation state of the promoter by using hypomethylating agents (see below).

## Immune evasion

The immune system has the ability to recognize and eradicate tumor cells and serves as a significant barrier to cancer development and progression [9]. Tumors hijack inhibitory checkpoints in order to evade immune eradication. In the recent years, checkpoint inhibition has revolutionized immunotherapy in cancer and delivered impressive outcomes for some patients with cancer [105]. However, selecting patients who are likely to respond to immunotherapy remains challenging. Hence, there is a critical need to better understand the mechanisms of immune evasion exploited by tumor as well as the cellular and molecular features of the tumor microenvironment in order to guide the development of rational and effective immunotherapy [10, 106].

### PD-1/PD-L1 pathway

In our previous gene expression profiling study, we observed upregulation of PD-ligand 1 (PD-L1, also known as CD274) mRNA in tumor compared to control tissues (GEO database GSE90597) [3, 8]. Several other studies have also reported overexpression of PD-L1 in ENKTL by immunohistochemistry [107–109]. Notably, oncogenic activation of the STAT3 pathway and overexpression of LMP1 induce the upregulation of PD-L1 in ENKTL, which may contribute to tumor escape from immune surveillance [110, 111]. Collectively, these data support the rationale of employing PD-1 checkpoint inhibition in the treatment of ENKTL. The first case series of seven patients with relapsed ENKTL and treated with pembrolizumab, an antibody against PD-1, was recently reported by Kwong et al. [11]. All patients experienced a rapid response to pembrolizumab, with five complete responses (median follow-up, 6 months). A similarly high efficacy was observed in two other studies using pembrolizumab and nivolumab in patients with relapsed or refractory NK/T cell lymphoma, indicating that PD-1 blockade is an important treatment strategy in ENKTL [112, 113]. However, the response to pembrolizumab did not show a definite correlation with immunohistochemical expression of PD-L1 expression in the neoplastic cells [11], indicating that protein expression of PD-L1 assessed by conventional immunohistochemistry may not be predictive of the response to checkpoint inhibition.

There is currently an unmet need for better predictive biomarkers for checkpoint inhibitor-based immunotherapy [114]. To this end, it is important to understand the differential expression of PD-L1 and PD-1 in neoplastic versus immune cells in the tumor microenvironment. Using double immunofluorescence, Nagato et al. demonstrated the positive expression of PD-L1 in CD68-positive macrophages in addition to CD56-positive tumor cells [115]. The presence of tumor-infiltrating immune cells showing morphologic features of macrophages has also been described in other studies [107, 108]. Since ENKTL tumor cells secrete interferon (IFN)-γ which is known to induce PD-L1 expression in macrophages, it is postulated that ENKTL may utilize the PD-L1/PD-1 pathway to inhibit immune suppression via the upregulation of PD-L1 expression either on tumor cells or on macrophages [115]. In contrast, PD-1 is expressed primarily in tumor-infiltrating lymphocytes, but not in tumor cells, with the reported frequency ranging from very low to 36% [107, 108, 115]. The majority of studies describing the upregulation of PD-L1 relied on single-marker immunohistochemistry and visual scoring of the staining intensity, which often suffers from interobserver and intraobserver variability. The application of multiplex immunohistochemistry and quantification using automated multispectral imaging platform provides the advantage of assessing both tumor and immune cell phenotypes and their spatial relationship, thus providing valuable and fundamental information necessary for the development of better cancer immunotherapy [114, 116].

### HLA dysregulation

The elimination of virus-infected cells is dependent primarily on a normal cytotoxic T lymphocyte (CTL) response in an MHC class I-restricted manner [117]. Based on the observation that several CTL-defined epitopes have been mapped to EBV latent membrane proteins (LMPs) restricted with HLA-A2, HLA-A11, or HLA-A24 antigen, Kanno et al. reported a significantly lower frequency of the HLA-A*0201 allele in Japanese patients with ENKTL compared to healthy controls and suggested that the HLA-A*0201-restricted CTL responses to LMPs in EBV-infected tumor cells may function in vivo to suppress the development of overt lymphoma [118]. In a subsequent first genome-wide association study of 189 ENKTL and 957 controls of Chinese descent, Li and colleagues revealed that the single nucleotide polymorphism (SNP) rs9277378 (located in HLA-DPB1) had the strongest association with ENKTL [119]. Since HLA-DP heterodimer is involved in extracellular antigen presentation to CD4-positive T cell lymphocytes, this finding suggests the importance of HLA-DP antigen presentation in the pathogenesis of ENKTL. Defects in the antigen processing and presentation serve as effective immune escape strategies and are associated with malignant transformation [120].

The use of antigen-specific CTLs recognizing and targeting EBV-associated viral antigens has been utilized as an immunotherapy for ENKTL [8, 66] with one study reporting complete response in four out of six ENKTL patients with active disease and continued complete response in five of five patients in a first or later remission cohort [121].

## DNA damage repair/genome instability

Maintenance of genomic stability is essential for cell survival, and an appropriate response to DNA damage is a critical part of this process. Deregulation of the DNA damage response (DDR) is a key feature and an enabling characteristic of cancer, allowing the accumulation of other hallmarks through mutations [9, 122]. Ataxia-telangiectasia mutated (ATM) and ataxia-telangiectasia related (ATR) kinases are key regulators of the DDR which are frequently deregulated in cancer [122]. The ATR protein was shown to be deregulated in a subset of ENKTL due to deletions resulting from aberrant splicing [123]. In other cases, ATR and related cell cycle checkpoint genes are overexpressed [7], reflective of the unique genomic instability phenotype in a given tumor. In our previous GEP study, many of the pathways and cellular processes enriched in genes differentially expressed between ENKTL and normal NK cells included cell cycle regulation and mitoses. There is a general enrichment of the DNA damage ATM/ATR regulation of G2/M checkpoint pathway, further strengthening the role for DNA damage and genomic instability in the disease pathogenesis [7]. Interestingly, one study provided in vitro evidence that resveratrol, a natural non-toxic phenolic compound found in the skin of grapes, produced anti-tumor effects by activating the DDR pathway in an ATM/Chk2/p53-dependent manner in ENKTL cell lines [124]. Furthermore, EBV LMP1 is known to inhibit the DDR pathways and DNA damage checkpoint activation, and may also play a role in promoting genomic instability in ENKTL [125]. p53 plays a crucial role in the DDR and the maintenance of genomic stability [122]. As discussed in the “p53” section, p53 is a deregulated ENKTL and may contribute further to genomic instability [69]. Further studies are necessary to clarify and characterize the functional and clinical relevance of DDR in ENKTL with potential implications for targeted therapy [122].

## Inducing angiogenesis

Vascularization of tumors is essential for their growth. Even with all the growth advantages, tumors cannot enlarge beyond 1 to 2 mm in diameter unless they are vascularized [126]. In view of the angiocentric growth pattern that is characteristic of ENKTL, it is tempting to speculate that the activation of angiogenesis pathways may play an important role in the pathogenesis of this neoplasm. Indeed, overexpression of genes related to angiogenesis have been observed in ENKTL as compared to other peripheral T cell lymphomas or normal NK cells. In particular, the expression of a well-known angiogenesis inducer vascular endothelial growth factor A (VEGF-A), and its receptor VEGFR2 (KDR), have both been demonstrated in ENKTCL tissues and cell lines at the mRNA and protein levels [5, 127]. In addition, overexpression of hepatocyte growth factor (HGF) as well as its receptor MET (a surface receptor with tyrosine kinase activity), a ligand-receptor pair that is linked to the stimulation of angiogenesis, has also been reported at the mRNA level in ENKTL cases [5]. These findings support the biological relevance of angiogenesis-related pathways in the pathogenesis of ENKTL, providing a rationale to investigate the therapeutic benefits of anti-VEGF antibody bevacizumab and small molecule MET inhibitors in ENKTL [128, 129]. One study analyzed the therapeutic potential of bevacizumab in combination with cyclophosphamide, doxorubicin, vincristine, and prednisolone (CHOP) in a small, heterogeneous group of patients with T cell lymphomas that included ENKTL cases, and observed only a modest overall response rate of 53% [130]. Further evaluation in larger clinical trials focusing on patients with ENKTL is necessary to firmly establish the role and therapeutic benefits of these angiogenesis inhibitors.

## Other pathogenic pathways and novel targets

This section includes specific targets or pathways which have been implicated in the pathogenesis, prognosis, or therapeutics of ENKTL, but the precise mechanisms are not well understood based on the current data or do not fit into the hallmarks of cancer pathways as proposed by Hanahan and Weinberg.

### EBV as a driver of NK cell malignancies

ENKTL is universally associated with EBV viral infection [2, 131, 132] and is characterized by a type II latency pattern with the expression of EBNA1 and LMP1 but absence of EBNA2 [2, 133, 134]. EBNA1 is expressed in all virus-infected cells and is required for the replication and maintenance of the viral genome [135] but may not have a crucial function in the direct immortalization process and the tumorigenicity of EBV [136]. LMP1, on the other hand, is a major EBV oncoprotein. It functions as a classic oncogene in a rodent cell-transformation assay [137] and is vital for EBV-induced B cell proliferation in vitro [138, 139]. Functionally, LMP1 acts as a constitutively activated member of the tumor necrosis factor (TNF) receptor superfamily, mimicking CD40 in vivo and activating downstream signaling pathways, including NF-κB and MAPK pathways, in a ligand-independent manner [140–144]. However, in contrast to EBV-infected B cells, in vitro studies on NK cell lines reported a limited role of LMP1 in direct transformation of the neoplastic cells [145, 146]. Instead, it was suggested that the role of LMP1 in NK and T cells is to increase the sensitivity of the infected cell to the growth-promoting effects of IL-2 [146]. EBV also mediates the deregulation of miRNAs, in particular the downregulation of let-7g, let-7a, and let-7c and upregulation of miR-155 which has oncogenic function. [147, 148] The mechanisms underlying the EBV-mediated miRNA deregulation in ENKTL are as yet incompletely understood, and further work is required to decipher the processes involved.

Given the close association of EBV infection and its potential importance in the pathogenesis of ENKTL, exploiting therapies that specifically target viral proteins appear to be a conceivable therapeutic approach. As mentioned above, CTLs directed at LMPs have shown efficacy in treating ENKTL patients [121]. Other therapeutic approaches include abrogating the effector functions of LMP1, either directly by using anti-sense RNA [149] or indirectly by targeting its downstream signaling molecules such as components of NF-κB pathway [150].

### CD38

CD38 is a glycoprotein belonging to a complex family of enzymes on the cell surface, and it plays a role in the catabolism of extracellular nucleotides [151]. Our previous GEP data have demonstrated the upregulation of the CD38 gene in ENKTL tumor compared to control tissues (GEO database GSE90597) [8]. Strong expression of CD38 protein is seen in 50% of ENKTL patients, and this is associated with an adverse outcome [152]. Daratumumab, the humanized monoclonal antibody which targets CD38, has been approved for the treatment of relapsed multiple myeloma. Its therapeutic role is being explored in other lymphoproliferative disorders, and a preliminary in vitro study reported good efficacy against ENKTL [153]. A recent case report of a patient relapsing after bone marrow transplantation with dramatic response to daratumumab monotherapy further suggests that CD38 may be an attractive therapeutic target in ENKTL [154]. Further studies are required to decipher the precise mechanisms of CD38-targeted immunotherapy in ENKTL.

### Potential pro-proliferative oncogenes

KIT mutations are key drivers of gastrointestinal stromal tumors and mastocytosis [155]. KIT mutations are reported in up to 52% of ENKTL cases; however, these do not lead to a gain of function [156]. Mutations in the RAS family of oncogenes (KRAS, NRAS) as well as the beta-catenin pathway (CTNNB1) have also been described in ENTKL [18, 71]. The role of these mutations in ENKTL lymphomagenesis requires further study.

### Epigenetic deregulation

BCL-6 corepressor (BCOR) and mixed lineage leukemia 2 (MLL2) are epigenetic regulators known to have a role in cancer. BCOR interacts with some histone-deacetylase-family genes, and MLL2 encodes a histone methyltransferase [157, 158]. Mutations of both genes have been reported in ENKTL and appear to be mutually exclusive [14, 18, 159]. The relevance of these mutations to the pathogenesis of ENKTL remains to be evaluated. Mutations in other epigenetic modifiers such as ASXL3, ARID1A, and EP300 have also been reported at varying frequencies in ENKTL, and their biological relevance remains to be determined [18].

## Conclusions and future directions

Major advances in our understanding of the biology of ENKTL have been made in the last decade. The identification of potential tumor suppressor genes, PRDM1 and PTPRK, along with pro-survival signaling via MYC and NF-κB is of particular importance. The discovery of transcriptional dysregulation through RUNX3 and non-canonical functions of EZH2 in ENKTL have provided novel insights into the molecular pathogenesis of this disease. Despite the biological significance of these findings, their clinical relevance remains to be established. From a therapeutic translational viewpoint, we propose that immune checkpoint inhibition is one of the most promising avenues for drug development in ENKTL [160].

Our understanding of the cell surface expression patterns of PD-1 and PD-L1 in ENKTL remains incomplete. Similarly, the intracellular regulation of PD-L1 expression in ENKTL requires investigation. The role of CMTM6 (a novel regulator of PD-L1) in ENKTL would be an interesting area for further research [161]. Checkpoint inhibition should be evaluated in both newly diagnosed and relapsed ENKTL patients in the context of clinical trials. Clinical trials evaluating JAK inhibition in ENKTL are currently underway, supported by in vitro evidence of the dysregulation of this pathway. Finally, we propose that CD38 may be a promising target for evaluation in clinical trials given its strong and uniform expression in ENKTL as well as promising early clinical data. The combination of anti-CD38 antibodies or JAK inhibitors with l-asparaginase-based chemotherapy in the up-front or relapsed setting may be a promising avenue to improve outcomes for these patients. How best to combine these treatments, what are the best combinations, and for which groups of patients will be critical questions of clinical importance moving forward. The current limitations of model systems to test these combinations will present a significant challenge.
